# Temporal dynamics of ocular torsion and vertical vergence during visual, vestibular, and visuovestibular rotations

**DOI:** 10.1007/s00221-024-06842-7

**Published:** 2024-05-02

**Authors:** Tobias Wibble

**Affiliations:** 1 Department of Clinical Neuroscience, Division of Eye and Vision, Marianne Bernadotte Centre, Karolinska Institutet, Stockholm, Sweden; 2 St Erik Eye Hospital, Stockholm, Sweden

**Keywords:** Ocular torsion, Vergence, Gaze stabilization, Eye tracking, Eye movements, Visuovestibular

## Abstract

Ocular torsion and vertical divergence reflect the brain’s sensorimotor integration of motion through the vestibulo-ocular reflex (VOR) and the optokinetic reflex (OKR) to roll rotations. Torsion and vergence however express different response patterns depending on several motion variables, but research on their temporal dynamics remains limited. This study investigated the onset times of ocular torsion (OT) and vertical vergence (VV) during visual, vestibular, and visuovestibular motion, as well as their relative decay rates following prolonged optokinetic stimulations. Temporal characteristics were retrieved from three separate investigations where the level of visual clutter and acceleration were controlled. Video eye-tracking was used to retrieve the eye-movement parameters from a total of 41 healthy participants across all trials. Ocular torsion consistently initiated earlier than vertical vergence, particularly evident under intensified visual information density, and higher clutter levels were associated with more balanced decay rates. Additionally, stimulation modality and accelerations affected the onsets of both eye movements, with visuovestibular motion triggering earlier responses compared to vestibular motion, and increased accelerations leading to earlier onsets for both movements. The present study showed that joint visuovestibular responses produced more rapid onsets, indicating a synergetic sensorimotor process. It also showed that visual content acted as a fusional force during the decay period, and imposed greater influence over the torsional onset compared to vergence. Acceleration, by contrast, did not affect the temporal relationship between the two eye movements. Altogether, these findings provide insights into the sensorimotor integration of the vestibulo-ocular and optokinetic reflex arcs.

## Introduction

Beyond its capacity for vertical and horizontal displacements, the eye readily rotates in the roll plane (Guyton 1988). This happens naturally with every step we take, as the head slightly tilts from side to side, causing an ocular counter-roll in the opposite direction of the head roll (Pansell 2003). This ocular torsion (OT), is almost always associated with a vertical divergence of the two eyes, with the eye ipsilateral to the head tilt moving upwards in relation to the contralateral eye (Pansell et al. 2003). The gain, i.e., the velocity of the eyes in relation to the movement of the head, is considerably lower for the torsional response compared to its vertical or horizontal dimensions with considerable differences between individuals (Pansell et al. 2003, 2005), and both OT and vertical vergence (VV) express a more dynamic response pattern than conjugated eye movements in the horizontal or vertical planes (Pansell et al. 2003; Wibble and Pansell 2019, 2020; Wibble et al. 2020a, 2020b). Recording and evaluating eye movement responses in the roll plane therefore allows for a finer assessment of how motion parameters impact gaze-stabilization.

Beyond their being triggered by head movements through the vestibulo-ocular reflex (VOR), OT and VV will be expressed in response to viewing a rotated visual scene through the optokinetic reflex (OKR) (Hardcastle and Krapp 2016; Straka et al. 2014). OT and VV consequently present a concrete and objective way to quantify how the brain integrates visual and vestibular information towards a reflexive sensorimotor response. This response can also be altered depending on the visual content; increased visual clutter will increase the relative gain of torsion over vergence (Wibble and Pansell 2019), while heightened accelerations will lead to an inverse shift (Wibble et al. 2020a). These eye movement responses are also affected by cognitive states such as alertness or attention (Frattini and Wibble 2021; Magnusson et al. 1985), and they have been shown to act as promising biomarkers for visual processing disorders following concussion (Wibble et al. 2023; Bertolini et al. 2020).

While the dynamic responses of these two eye movements are becoming increasingly described, little research has been done on their temporal dynamics. One experimental protocol showed that the time to peak amplitude for OT and VV were 160 ± 30 ms (SD) for both eyes (Pansell et al. 2003). There has however been very little research performed with regards to the onset times of the two eye movements. Due to the orbital dynamics of the eyes, we may expect that OT and VV accompany each other, both due to the pulling directions of the extraocular muscles and the pulley system (Guyton 1988; Demer 2002), and the physiological deviations in the centre-of-rotations that may exist between the two eyes in relation to Listing’s plane (Furman and Schor 2003). We nevertheless do not know if the two eye movements start in unison, or if their onset times will shift depending on their method of induction, i.e., visual or vestibular, or how they may be affected by motion acceleration or the presence of visual information. After the motion has stopped we may also expect that the eyes will return to a baseline, and that the torsional and vertical position must decay over the course of milliseconds or seconds. The decay rate for visually induced ocular torsion was in one study outlined as approximately 1.10s, given the particular parameters of that investigation (Mezey et al. 2004), but there is little research done on the relative decay rates between OT and VV and how they may be affected by visual content.

The present study aims to explore the temporal dynamics of ocular torsion and vertical vergence in terms of their onsets during visual, vestibular, and visuovestibular motion, evaluating the response time for the sensorimotor integration of the vestibulo-ocular and optokinetic reflexive arcs. The study also outlines the relative decay rates of the eye position following prolonged optokinetic stimulations. This was carried out by collating temporal data from three separate investigations where participants’ eyes were tracked using video eye-tracking during a range of visual and vestibular movements, shedding further light upon the relationship between ocular torsion and vertical divergence from a broad neurophysiological perspective.

## Material and methods

The present study investigated the temporal dynamics of ocular torsion and vertical vergence in terms of their relative onset times as triggered by visual, vestibular, and visuovestibular motion. Their respective response times were furthermore assessed with respect to the amount of visual information density, i.e. clutter, in a viewed optokinetic scene, and the acceleration of the stimulus. This allowed us to investigate if the aforementioned factors impacted the onset times of the two eye movements as well as their temporal dynamics in relation to one another. The decay rates of the torsional and vergence eye position following prolonged optokinetic rotations were also assessed.

The present study collated data retrieved from three separate trials investigating the eye movement responses of ocular torsion and vertical vergence to visual, vestibular, and visuovestibular motion. Results from these studies have been previously presented in terms of slow-phase gain of each eye movement. However, no data concerning the temporal dynamics of the torsional and vergence responses has been published. The present study made use of these datasets to allow a comprehensive statistical framework, allowing a higher statistical power than those retrieved for the isolated investigations.

### Participants

Investigation One (Wibble and Pansell 2019) included 12 healthy subjects, 8 male and 4 female, with an average age of 43 years (ranging from 25 to 72). Thirteen individuals, comprising 7 men and 6 women with an average age of 25 years (ranging from 23 to 34), were recruited as volunteers for Investigation Two (Wibble et al. 2020a). Investigation three (Wibble et al. 2020b) included 16 healthy participants, 8 men and 8 women (ranging from 19 to 65 years of age). Subjects were recruited according to the same criteria for all investigations: None of the participants had any medical conditions or used drugs known to affect the central nervous system. All demonstrated normal or corrected visual acuity (VA) of at least 1.0 using the logarithm of the minimum angle of resolution (logMAR chart), stereoscopic vision of at least 200″ of arc (tested with the Lang II stereotest), and normal eye movement. Before participation, any considerable latent strabismus was ruled out using the cover test. None had a history of vertigo, and vestibular function was evaluated using a horizontal head impulse test, which showed no refixation saccade. Balance was assessed using the Romberg’s test on a soft platform. All participants could reliably fixate their eyes on a viewed target, and maintain gaze position in darkness, as evaluated using a head mounted eye-tracker (see eye and head movement recording).

Before enrolling in the study, all participants provided informed written consent after receiving detailed explanations about the study’s nature, along with both written and oral information about the procedure. The research adhered to the principles outlined in the Declaration of Helsinki and received approval from the Regional Ethics Committee of Stockholm (EPN 2018-1768-31-1).

### Experimental setup

**Fig. 1:**
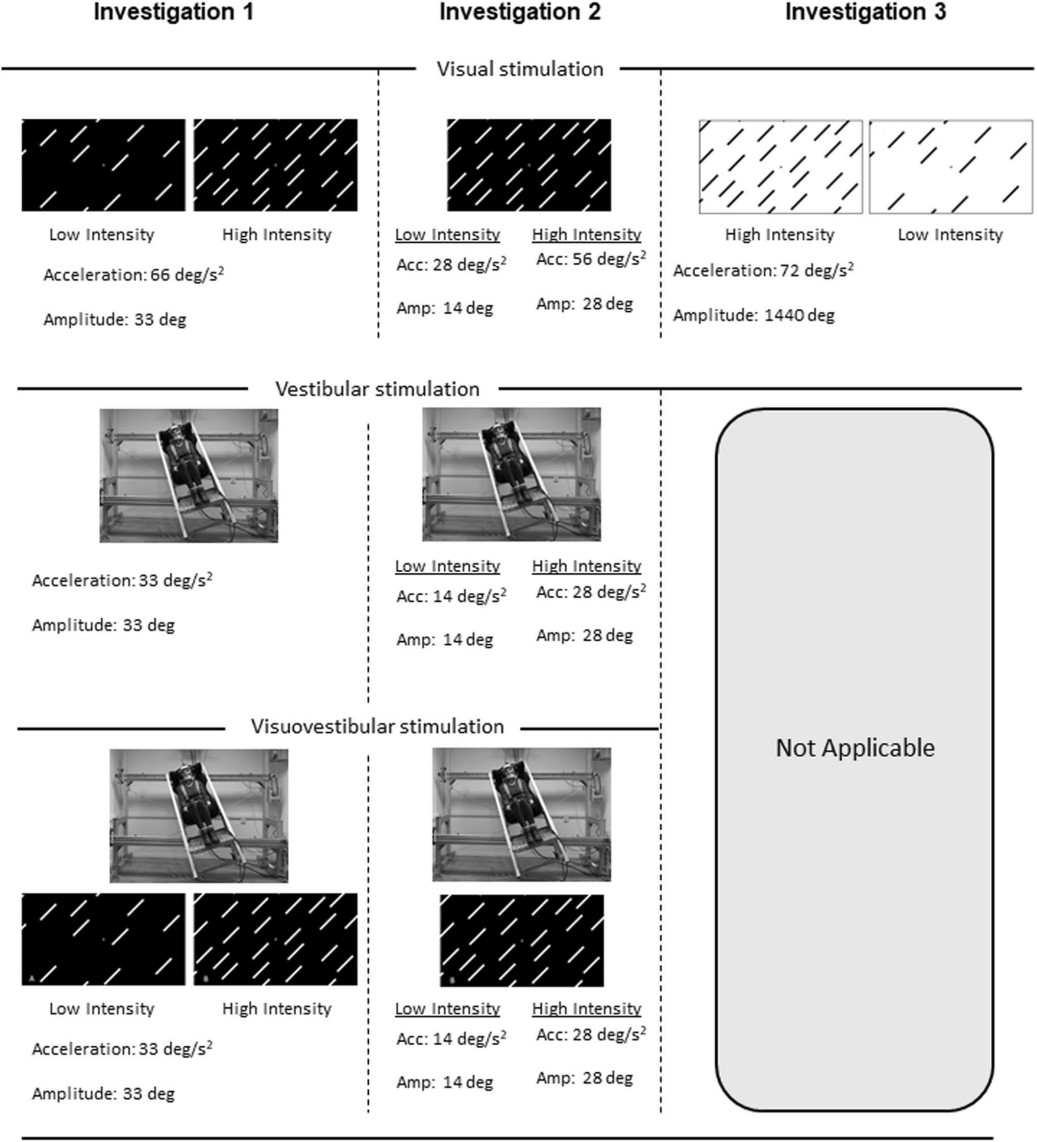
The experimental setups of the investigations included in the present study. Investigation one and two featured visual, vestibular, and visuovestibular trials, whereas investigation three only featured prolonged optokinetic stimulations. The acceleration (acc) and amplitude (amp) of each stimulation are given in the corresponding column and row. The level of intensity (low or high) refers to the order of magnitude for each variable (visual clutter or acceleration level)

Investigations one and two implemented a protocol evaluating torsional and vergence eye movements during visual, vestibular, and visuovestibular rotations in the roll plane; Investigation one implemented two different levels of visual information density in the viewed visual scene, i.e., visual clutter, while investigation two issues all stimulations at two different accelerations. These stimulations were carried out with durations of only a few seconds. By contrast, investigation three exposed subjects to visual rotations for a duration of 20 s, allowing a greater displacement of the eye position; these trials were repeated using the two different levels of visual clutter as first implemented in investigation one. Altogether, the present investigation collated the temporal dynamics retrieved from these three investigations, allowing us to explore how torsional and vergence latencies are affected by acceleration, visual clutter, and modalities, i.e., visual, vestibular, or visuovestibular motion. The sensory-specific protocols are outlined below, and can be found summarized in Fig. 1. All participants were exposed to all trials within each investigation.

### Visual stimulations

All visual stimulations were carried out on a projected screen (res 1024 × 768; contrast 2000:1; update frequency 60 Hz) by a front video projector (NEC NP-M350X, NEC Display Solutions Ltd., Tokyo) presented at an eye-screen distance of 200 cm. Visual elements consisted of white lines on a black background for investigations one and two, and black lines on a white background for investigation three, presenting comparable visual contrasts. The low intensity visual clutter featured 19 lines while the high intensity held 38 lines, all being viewed at a visual angle of 0.93 degrees. Investigation one and three used both these clutter levels, while investigation two used only the high intensity. These clutter levels always provided the same lighting levels during the stimulation, as tested using a photometer (universal photometer model S4; Hagner, Solna, Sweden).

Investigation one featured an optokinetic acceleration of 66 deg/s^2^ to an amplitude of 33 degrees, investigation two presented stimuli at 28 deg/s^2^ to an amplitude of 14 degrees and 58 deg/s^2^ to an amplitude of 28 degrees, and investigation three’s stimulation moved at 72 deg/s^2^ for 1440 degrees. Investigation one and two featured subjects seated, while subjects were standing during investigation three. Participants were tasked to always fixate on a high-contrast fixation point centred in the visual scene during each trial; these started with 20 s of baseline during which time the static visual scene was presented, after which the active motion was initiated. After the termination of motion, all subjects retained fixation for another 20 s until the recording was stopped. Investigations one and three featured optokinetic motion in both clockwise and counter-clockwise directions, but as no effect of direction was seen only counter-clockwise motion was implemented during investigation two.

### Vestibular stimulations

All isolated vestibular stimulations in investigations one and two were carried out by rotating participants using an in-lab constructed mechanized sled which rotated on two belts powered by two AC Brushless Servo Motors (Baldor BSM90C, 400 V) in a dark room. Light pollution was precluded by framing the room with blackout curtains. To minimize darkness adaptation, participants were also exposed to an intensive bright light 5 s prior to the start of recording, after which a baseline was established during 20 s before the movement was implemented. Vestibular trials were only implemented during investigations one and two and were carried out at the same amplitudes but at half the acceleration of their respective visual counterpart. The centre-of-rotation was set to the glabella for all subjects in order to adjust for differences in height.

### Visuovestibular stimulations

Visuovestibular trials were carried out according to the same principle as the vestibular stimulations, i.e., through whole-body rotations of each participant, but with subjects viewing the static visual scene associated with each respective investigation. This means that investigation one featured visuovestibular trials where subjects were rotated while viewing both low- and high intensity visual clutter. Investigation two instead introduced only the high intensity visual clutter but rotated at two different accelerations. This meant that subjects were exposed to the summated motions of the visual and vestibular trials of each investigation.

### Eye- and head recording

Eye movements were tracked using the head-mounted Chronos Eye Tracker Device (C-ETD; Chronos Inc, Berlin, Germany). This system was designed for binocular recordings, and the recording rate was 100 Hz with high spatial resolution of < 0.05° for horizontal and vertical eye movements, and < 0.1° for ocular torsion. The torsional movements were quantified as rotational displacements of the iris around the centre of the pupil by tracking iris features using the integrated Chronos software. This technique relied on template matching each frame with the initial reference frame obtained at the start of recording. Through cross-correlation, where a value of 1.0 indicated perfect matching, each frame was assigned a quality measure ranging from 0 to 1.0. Frames with a quality value below 0.5 were excluded from the analysis.

The horizontal and vertical displacements of the pupil were initially calibrated by instructing the participant to perform a series of eye movements to a pattern of dots with known separations, thereby allowing for the conversion of pupil displacement into angular degrees of horizontal, vertical, and torsional eye rotations. As video-based systems have been shown to be susceptible to iris occlusions, uneven lighting conditions, or poor pupil definition, multiple iris segments were collected for analysis, with each segment evaluated to determine the highest possible signal quality. Furthermore, all measurements were conducted under uniform lighting conditions, with the room darkened using blackout cloth and the projector serving as the sole light source. The tracked iris region was positioned away from any corneal light reflexes or lid shadows that could potentially interfere with the signal. In the event that the eye position signal was disrupted due to blinking during stimulation the trial was repeated.

A head tracking system was incorporated into the headgear for simultaneous recording of head movements in six dimensions (three rotational and three translational). This enabled precise measurement of head movements, ensuring that the subject remained stationary or moved at a precise rate in accordance with the vestibular or visual stimulation requirements. Head movements were monitored in all trials to ensure that no confounding activation of the VOR would influence the eye movement response. No such confounding head movement was seen for any trial.

#### Analyses

**Fig. 2:**
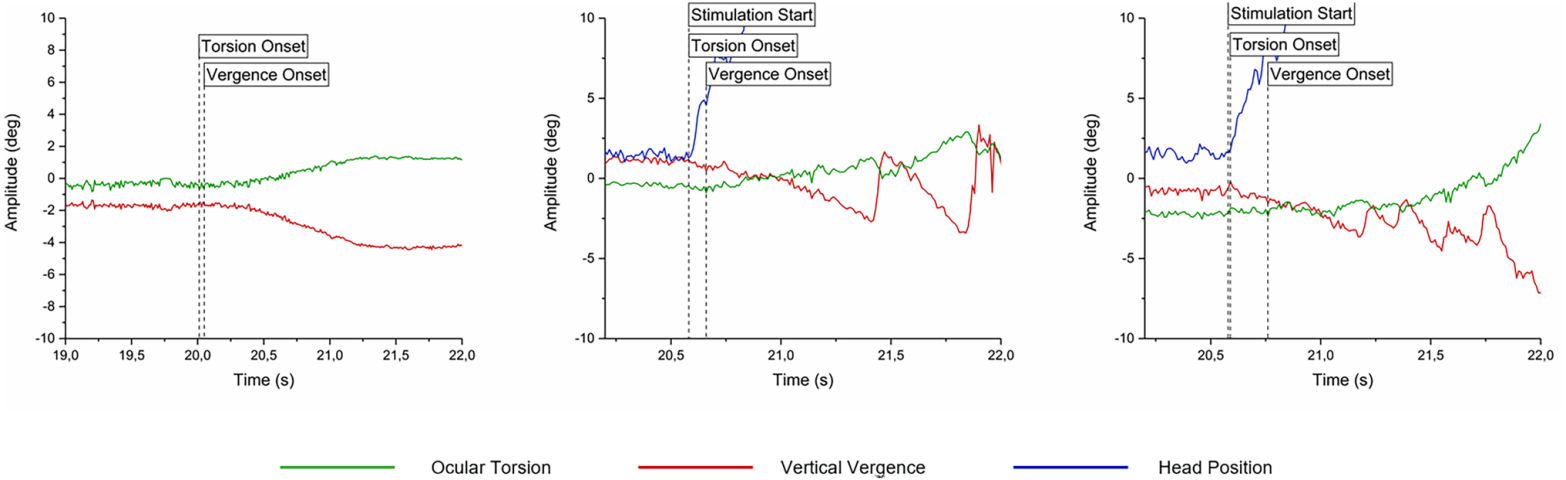
Representative traces illustrating the torsional and vergence responses at the start of visual, vestibular, and visuovestibular motion. The onsets of the vestibular and visuovestibular trial are highlighted by the head position indicating the stimulation start. The torsional and vergence onsets are indicated by the dotted lines

The recorded sequences were subjected to processing using the analysis software integrated with the eye-tracking system (Chronos Vision GmbH, Berlin) to derive values for horizontal and vertical pupil positions, as well as torsional displacement of iris position, in degrees. The vertical skewing response was computed by subtracting the vertical eye position of the left eye from that of the right eye, while the torsional response was determined based on the eye with the most favourable signal-to-noise ratio. The retrieved data was plotted graphically in the Origin software (OriginPro 2017; OriginLab, Northampton, MA). An investigator manually evaluated all traces to determine the onset of both the torsional and vergence response. These were determined on the basis of presenting a clear deviation from the baseline in the direction corresponding to the movement stimulation. Examples of the torsional and vergence onsets can be found in Fig. 2. The decay rate of the eye position for investigation three was determined using the Origin software’s exponential decay function (ExpDecay1; y = y0 + A1*exp(−(x–x0)/t1)), and the time constant was retrieved. This was done for the time period immediately following the visual stimulation, i.e., between 40 and 60 s of the recording.

Stimulation triggers were performed manually, meaning that there was a margin of error for the onset of the stimulation itself; the stimulation and the eye-movement recording were triggered through different programs, and both were started by the same investigator simultaneously pressing two triggers. This created uncertainty in calculating the latencies for torsional and vergence eye movements in response to visual stimulations, as the start of visual motion could not be determined with high enough precision. Thanks to the had-mounted accelerometer, the vestibular and visuovestibular responses could be determined in relation to the start of the head movement with high temporal precision.

Statistical analyses were carried out according to two principles: (1) First level analyses were carried out within each investigation to allow for within-subject analyses where appropriate. These were carried out using repeated measures ANOVA in JASP (Version 0.16.4; JASP Team 2019, which was also used to perform test of normality. In the case of non-normal distribution or missing data points, a Generalized Linear Model (GLM) was implemented using SPSS Statistics 25 for Windows. (2) Data from investigations one and two were collated into one GLM, allowing a thorough evaluation of the onsets of ocular torsion and vertical divergence; note that investigation three was excluded from this model as it presented a different method of stimulation as outlined in Fig. 1. α was set to 0.05.

## Results

### Optokinetic responses

An initial repeated measures ANOVA revealed a significant difference between torsional and vergence latencies during visual rotations at different accelerations (*F* [1, 12] = 42.109; *P* < 0.001; partial η^2^ = 0.778). There was no effect of acceleration on the eye movement response onsets, indicating that the relationship between torsion and vergence was not affected by optokinetic accelerations.

Eye movements similarly exhibited significantly different onsets in comparable trials presenting two different versions of visual clutter (*F* [1, 10] = 5.899; *P* = 0.036; partial η^2^ = 0.371). Torsion and vergence responses were significantly affected by clutter levels (*F* [1, 10] = 4.872; *P* = 0.052; partial η^2^ = 0.328), where higher amounts of visual content increased the duration between their onsets, with the torsional response being recorded earlier and the vergence movement later.

This finding was also seen when comparing torsional and vergence onsets during the prolonged optokinetic viewing; there was a significant difference between the torsional and vergence response (*F* [1, 15] = 23.412; *P* < 0.001; partial η^2^ = 0.609), and this ratio was affected by clutter levels in the same fashion as outlined above (*F* [1, 15] = 7.951; *P* = 0.013; partial η^2^ = 0.346).

The prolonged optokinetic stimulation caused the eye to build up a significant amplitude over the course of 20 s, allowing us to evaluate the decay rate of the torsional and vergence positions. Not all trials lead to a significant shift in amplitudes large enough to allow the quantification of the decay rate, leading to eight missing data points for the vergence response and five for torsion. A generalized linear model was used to account for this fact, comparing the difference between torsional and vergence onsets to low- and higher levels of visual clutter. This showed that higher levels of clutter significantly decreased the differences in torsional and vergence decay rates (*X*^*2*^(2, N) = 5847, p = 0.016). There was no significant difference between torsion and vergence decay rates if not accounting for clutter levels.

### Vestibular responses

The ANOVA investigating the effects of acceleration on the onsets of ocular torsional and vertical vergence during vestibular stimulations in darkness showed a significant difference between the two eye movements (*F* [1, 12] = 5.328; *P* = 0.040; partial η^2^ = 0.307), indicating that the torsional response was seen before its vergence counterpart.

### Visuo-Vestibular responses

The Visuovestibular onsets failed to meet the assumption of normality for both torsion and vertical vergence, for which reason a GLM was implemented, using eye movement and clutter levels as predictors and factors. This model showed a significant difference between the two eye movements (*X*^*2*^(1, N) = 7733, p = 0.005), and similar to both visual and vestibular modalities showed that the torsional response preceded the vergence movement.

### Collated analysis

A single GLM model was used on the unified data from investigations one and two to allow a unified model evaluating the onsets of ocular torsion and vertical divergence; investigation three was omitted due to its different stimulation protocol (see Fig. 1) This model implemented acceleration, visual clutter levels, modality, and type of eye movement as predictors as well as factors. Like all previous models, there was a significant difference between the two eye movements (*X*^*2*^(1, N) = 4260, p = 0.039). There was also a significant difference between modalities (*X*^*2*^(1, N) = 12,274, p < 0.001), and between motion accelerations (*X*^*2*^(4, N) = 16,599, p = 0.002), where increased accelerations were generally associated with earlier onsets of the two eye movements. This means that the torsional onset was seen before the vergence eye movement, and that the average onset time was seen earliest for visual stimulations, and latest for vestibular stimulations. However, due to the uncertainty concerning the onset of the visual stimulation it is impossible to ascertain the correct eye movement starting times with the required temporal resolution. Owing to the head accelerometer data the results still allow us to conclude that the visuovestibular onsets preceded the vestibular responses; this was supported by a GLM on eye movement latencies in relation to vestibular and visuovestibular stimulations, including eye movement type and modality as predictors and factors (*X*^*2*^(1, N) = 12,972, p < 0.001).

The model did not allow for evaluating interaction effects due to the discrepancies between accelerations and other factors; visual accelerations were carried out at 28, 36, and 66 deg/s^2^ compared to 14, 28, and 33 deg/s^2^ for vestibular and visuovestibular trials, leading to inadequate points of reference for the statistical model. In order to evaluate possible interactions effects, a GLM precluding acceleration as a factor albeit including it as a predictor was implemented, testing the difference between torsional and vergence onsets as the dependent variable. This model showed a significant interaction effect between clutter levels and stimulation modalities (*X*^*2*^(1, N) = 4098, p = 0.043). This indicated that amplified visual clutter increased the differences between the torsional and vergence onsets during optokinetic viewing compared to the visuovestibular stimulations and the vestibular trials in darkness.

### Summary of main findings

**Fig. 3:**
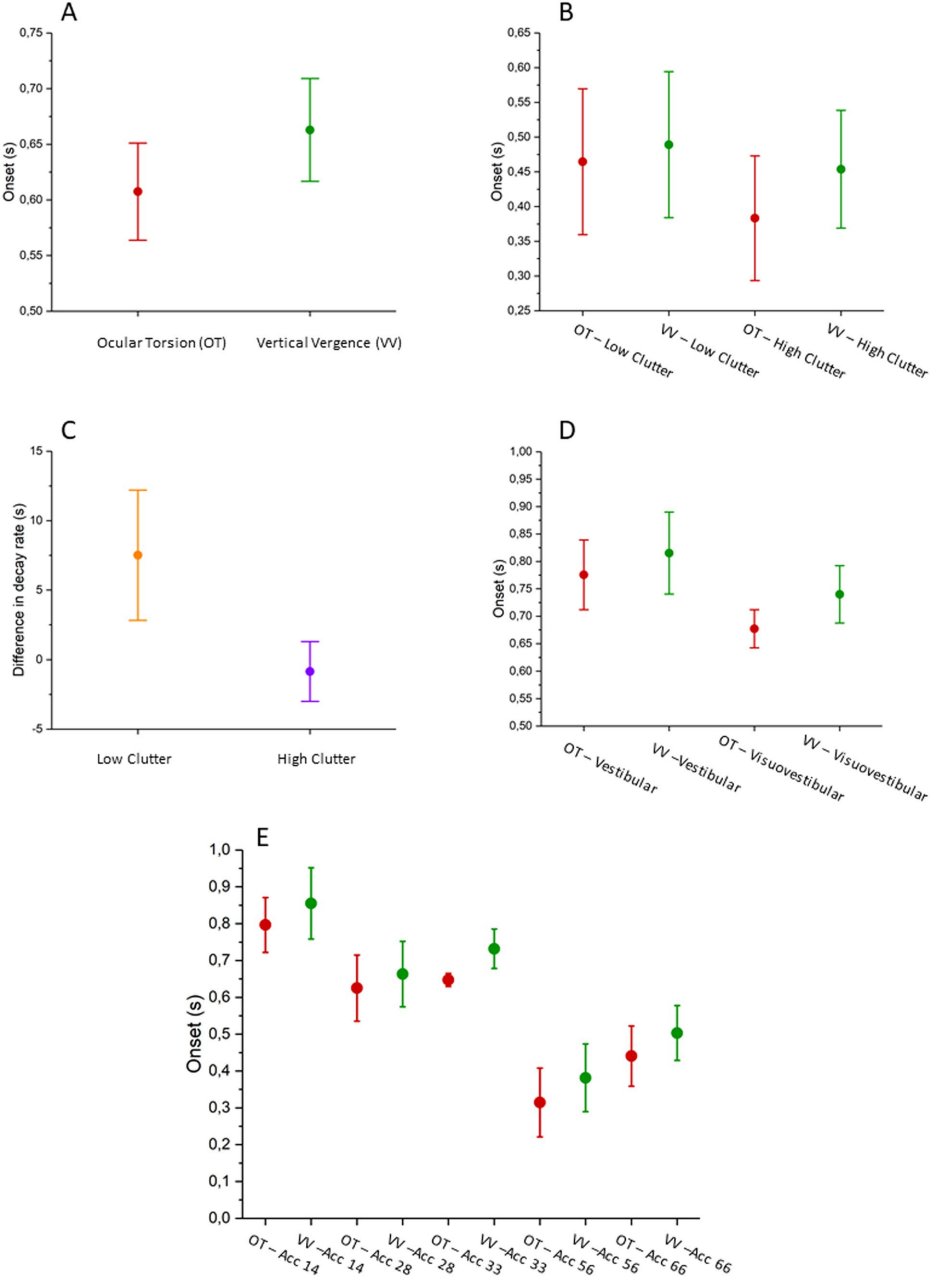
Interval plots showing the mean onset times (± 95% confidence interval) of ocular torsion (OT) and vertical vergence (VV) across all trials (A; (*X*^*2*^(1, N) = 4260, p = 0.039)), and depending on visual clutter intensities (B; (*X*^*2*^(1, N) = 4098, p = 0.043)). The difference in decay rates between OT and VV was similarly impacted by clutter levels (C; (*X*^*2*^(2, N) = 5847, p = 0.016)), and the onset times seen earlier during visuovestibular trials compared to vestibular motion in darkness (D; (*X*^*2*^(4, N) = 16,599, p = 0.002)) where increased motion accelerations were generally associated with earlier onset times for both OT and VV

Altogether, these findings show that torsion exhibits and earlier onset than vertical vergence (see Fig. 3A). The difference between these onsets were increased when the amount of visual information density was intensified (see Fig. 3B). Clutter levels also had a significant effect on the decay rates of the torsional and vertical eye positions, where an increased level of clutter was associated with a more balanced rate of decay (see Fig. 3C).

**Fig. 4:**
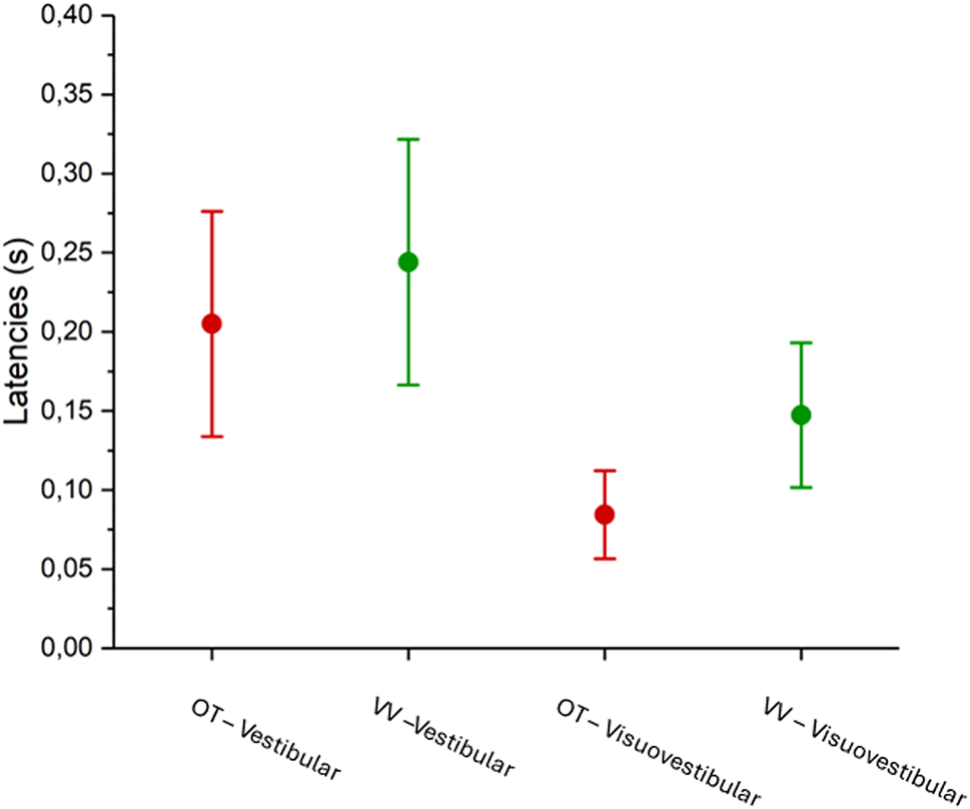
A descriptive box plot showing mean latencies (± 95% confidence interval) for ocular torsion (OT) and vertical vergence (VV) during vestibular and visuovestibular trials. The values were retrieved in relation to the stimulation start as indicated by the head-mounted accelerometer

Torsion and vergence onsets were similarly affected by stimulation modality and accelerations; both torsional and vergence onsets were seen earlier for visuovestibular motion compared to vestibular motion in darkness (Fig. 3D) while increased accelerations were associated with earlier onsets for both eye movements regardless of modality (see Fig. 3E). However, the findings concerning effects of modality warrant further discussion due to the uncertainty in optokinetic trigger times. The results presented in Fig. 3 should therefore be viewed as indicative of the relative temporal dynamics between OT and VV rather than as absolute onset times in relation to the triggering stimulus. Owing to the head movement data recorded through the C-ETD the latencies between stimulation and eye movement response could be readily ascertained for vestibular and visuovestibular trials (see Fig. 4), presenting true onset times.

## Discussion

Ocular torsion and vertical vergence share important fundamental neurophysiological properties. This was also exemplified in the present study, showing that both eye movements are similarly impacted by their modality of induction and the motion acceleration. The visual stimulation further exemplified that the two eye movements involve separate sensorimotor mechanisms, as both onsets and decay rates were influenced by the levels of visual clutter.

The vertical divergence seen to optokinetic rotations has traditionally been viewed as secondary to ocular torsion and cyclovergence (Schor et al. 2002). As its distinct eye movement, visually induced vertical vergence may be considered a relatively newly described phenomenon. We have in a series of studies shown that the dynamic gain of visually induced VV increased in relation to its torsional counterpart with increased optokinetic accelerations (Wibble et al. 2020a), and decreases when there was higher amounts of visual clutter (Wibble and Pansell 2019); data from these investigations were also implemented in the present study. VV is also increased during monocular viewing compared to binocular conditions, meaning that it likely serves a fusional purpose as it is supressed during binocular viewing (Wibble and Pansell 2020). We have also described how visually induced VV is affected in concussed patients suffering from vestibular symptoms of vertigo or dizziness to visual motion (Wibble et al. 2023; Frattini et al. 2024). The relationship between torsion and vergence during vestibular movements appears to remain relatively constant by comparison (Wibble and Pansell 2019; Wibble et al. 2020a). There consequently appears to be some elements to the sensorimotor integration of visual motion information that influences OT and VV differently. This was furthermore exemplified in the present study, where increased visual clutter increased the latencies between torsional and vergence onsets, while also decreasing the differences in their decay rates.

Concerning the increased difference in onset times, the present study showed that the torsional response arrived earlier when the clutter level was increased, while the vergence response was seen relatively later. This may be considered in line with ocular torsion previously being described as more sensitive to visual information density compared to vertical vergence (Wibble and Pansell 2019; Wibble et al. 2020b), and by collating data from two separate investigations the present study adds that this responsiveness to visual content is also reflected in the response time. The decay rate was analysed through a GLM as there were missing data points due to not all trials producing a significant enough amplitudal shift to allow for the decay constant to be calculated. This was more so the case for VV compared to OT, which may be expected due to VV presenting lower amplitudes compared to OT (Wibble et al. 2020b). The decreased difference in the decay rates may be attributed to the additional visual content that may aid in visual refixation. We have shown that VV likely serves a purpose for binocular fusion (Wibble and Pansell 2020), and the addition of visual content may aid the neural processes responsible for this fusion. This is further exemplified by previous research having shown that visual patterns that cannot be fused fail to produce cyclovergence, while still resulting in cyclotorsion (Rijn et al. 1992). It has been shown that vertical disparity is used to establish a framework for stereopsis (Mitsudo et al. 2013). While the visual scenes presented in the present study did not introduce visual disparities, the vertical misalignment caused by the rotation may contribute to a similar phenomenon, allowing for additional visual substrate that may speed up the process of fixation. One may hypothesize that the vestibular reference points present in the visuovestibular trials may offer stronger influences for refixation than the visual content, mitigating the effects of clutter levels.

The neurophysiological principles guiding the different response patterns of OT and VV to optokinetic motion remain unknown. Ocular torsion stems from the vertical gaze-centre at the interstitial nucleus of Cajal (Crawford et al. 1991), while the neural control of vertical divergence remains unspecified. Gaze-stabilizing responses can be influenced by a range of cognitive and cortical mechanisms (Angelaki and Cullen 2008), but fundamentally rely on a few subcortical nodes (Wibble et al. 2022). The vestibular nucleus, while dedicated to reflexive vestibular responses, also receives projection from pretectum which relays optokinetic information (Wibble et al. 2022). We have previously put forward that visually induced vertical vergence may primarily reflect a visual activation of this pretectal-vestibular relay, as VV is more responsive to typically vestibular factors such as acceleration (Wibble et al. 2020a), and is clinically considered indicative of vestibular pathologies or signalling in the form of skew deviation (Brandt and Dieterich 1993). The present findings fit well within such a theoretical framework, as increased visual information in the viewed scene moved the torsional onset forward in relation to the vergence response, supporting the existing evidence suggesting that torsion is more readily influenced by optokinetic content (Wibble and Pansell 2019; Wibble et al. 2020b) whereas vertical vergence appears to be visually supressed (Wibble and Pansell 2020).

The integration of isolated versus combined sensory inputs and its implications for sensory processing is a diverse and growing field of research. Response times during isolated sensory stimulations tend to be quicker to visual stimuli compared to auditory or tactile cues (Wang et al. 2012); Combined visuotactile cues produce even faster response times through multisensory facilitation (Wang et al. 2012). The addition of multiple senses is nevertheless not always beneficial for an individual’s reaction time, and the same study noted that the addition of auditory cues instead delayed the reaction onset (Wang et al. 2012). This divergent effect of multisensory integration can be dependent on the task at hand, and combined audiovisual stimuli may enhance subjects’ performance in terms of speed, but when faced with a discrimination task the auditory component may be largely neglected (Sinnett et al. 2008). The facilitating effects of receiving multiple inputs may be largely perception, relying on rapid subcortical networks, as compared to cognitive processes. The capacity to discern motion can for example be enforced through congruency between visual and auditory directional cues, while this ability instead is diminished if the two senses present diverging vectors (Kim et al. 2008). In this context the present findings provide further evidence that multisensory facilitation may occur on a subcortical basis with congruent visual and vestibular input, highlighting the synergistic properties of the optokinetic and vestibulo-ocular reflexive arcs.

While the present study also investigated the effects of acceleration on the temporal dynamics of OT and VV, the analysis was limited in that the optokinetic accelerations were issued at twice those of the vestibular and visuovestibular modalities. It is therefore somewhat precarious to conclude whether the modality or the acceleration serves as a driving factor in how the onset times were affected; were the earlier onsets seen to visual motion due to their sensitivity to optokinetic input, or was it because the acceleration was overall higher in the visual trials? However, the GLM remains a robust statistical model that to a certain extent may be expected to account for these discrepancies, and no interaction effects between modality and acceleration was identified. Furthermore, the difference between vestibular and visuovestibular conditions may not be attributed to acceleration levels, adding credence to the finding that both modalities and acceleration may have a significant effect on the onset times. While the true latencies of the optokinetic responses were not possible to determine due to the limitations in how the stimuli were triggered, results show that visuovestibular onsets arrived earlier than those observed during isolated vestibular stimulations. It consequently appears that there is a summative effect of OKR and VOR responses. The dynamic gain of the roll plane OKR and VOR has been described to express robust additive properties, where the visuovestibular gain equates the summated velocities seen during isolated visual and vestibular trials (Wibble and Pansell 2019; Wibble et al. 2020a, 2022). Contextualizing these studies with the present findings, this study suggests that OT and VV may exhibit faster temporal processing through the OKR, while the VOR more readily processes spatial information. The present study is significantly limited in its lack of outlining trigger times for the visual stimulations, making it impossible to discern eye movement latencies in relation to the onset of the stimulation and further studies would benefit from a more rigorous protocol for issuing visual stimuli. It may also be noted that the glabellar centre-of-rotation implemented in the current study was chosen to reflect a natural head tilt, as compared to rotations centred on the naso-occipital axis which instead is centred on the vestibular system (Diamond and Markham 1983). The latter may therefore be a more suitable choice as a centre-of-rotation for future studies aiming to discern the specifics of the distinct parts of the peripheral vestibular apparatus, as it may allow greater control over which semicircular canal is being manoeuvred at any given time.

## Conclusion

In summary, the present study delineates distinct patterns in the onset and decay rates of torsional and vertical vergence eye movements under varying visual and environmental conditions. Notably, torsion manifests an earlier onset compared to vertical vergence, a distinction that is exacerbated with heightened visual information density. Moreover, clutter levels exert a discernible influence on the decay rates of both torsional and vertical eye positions, with increased clutter associated with a more balanced rate of decay. Stimulation modality and accelerations further impact the onset of eye movements, with visuovestibular motion eliciting earlier responses compared to vestibular motion in darkness. While increased accelerations prompt earlier eye movement onsets, uncertainties in optokinetic trigger times necessitate further exploration. Altogether, these findings suggest synergistic temporal processing of joint visuovestibular motion information in the roll plane, and add further evidence to ocular torsion and vertical vergence relying on different neural pathways.

## Data Availability

Data will be made available upon reasonable request.
